# Variations of oral anatomy and common oral lesions^⋆^

**DOI:** 10.1016/j.abd.2023.06.001

**Published:** 2023-09-16

**Authors:** Paulo Ricardo Martins Souza, Letícia Dupont, Gabriela Mosena, Manuela Lima Dantas, Lucas Abascal Bulcão

**Affiliations:** a Dermatology Service, Hospital da Santa Casa de Misericórdia de Porto Alegre, Porto Alegre, RS, Brazil; b Department of Internal Medicine/Dermatology, Universidade Federal de Ciências da Saúde de Porto Alegre, Porto Alegre, RS, Brazil

**Keywords:** Mouth, Mouth mucosa, Mouth diseases, Pathology, oral

## Abstract

Several topics related to the oral cavity are briefly addressed in this article, from anatomical variations that, when recognized, avoid unnecessary investigations, to diseases that affect exclusively the mouth, mucocutaneous diseases, as well as oral manifestations of systemic diseases. A complete clinical examination comprises the examination of the mouth, and this approach facilitates clinical practice, shortening the path to diagnosis in the outpatient clinic as well as with in-hospital patients. The objective of this article is to encourage the examination of the oral cavity as a useful tool in medical practice, helping to recognize diseases in this location.

## Introduction

Oral diseases are an important public health problem and have a high prevalence.^1^ They affect all age groups and can be chronic and progressive, causing great negative impact on quality of life.^2^

The oral cavity must be evaluated as a whole, and it is important that the examiner standardizes the evaluation routine. Examination of the mouth comprises the vestibule (the part between the labial mucous membranes and the teeth), the inner part of the cheeks, the palate, the dorsum of the tongue, and the floor of the mouth, which, when examined, also allows the observation of the ventral part of the tongue, and the oropharynx. All of these regions should be examined and palpated, as well as the parotid, buccal, sublingual, submental, superficial, and deep cervical lymphnode chains.^3^

Anatomical variations of the mouth are extremely common and a frequent reason for clinical consultation and will also be discussed here. Some of them are present in more than 80% of the population^1^ and only require patient guidance. Recognizing its clinical aspects is essential to avoid unnecessary treatments or investigations.

On the other hand, oral alterations may suggest mucocutaneous or systemic diseases and should be part of the clinical reasoning as they may shortcut the investigation when they are recognized.

## Anatomical variations

### Fordyce granules

They are an extremely common anatomical variation. These are ectopic sebaceous glands that occur in the labial semi mucosa and oral mucosa. They usually appear in adulthood.^4^

**Figure 1:**
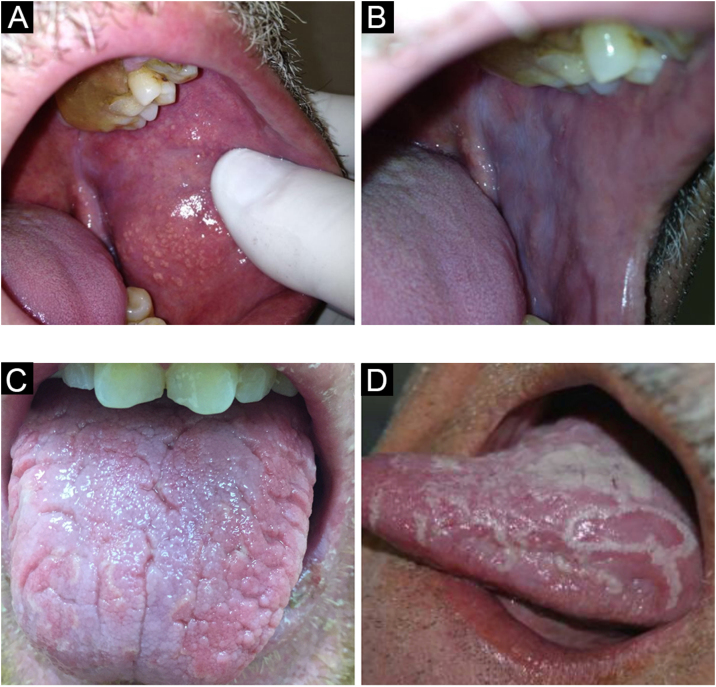
(A) Fordyce granules. (B) Leukoedema. (C) Fissured tongue. (D) Geographic tongue

They present as yellowish, round or polygonal, juxtaposed or isolated granules, usually in large number (Fig. 1A). True whitening of the upper lip semi mucosa, the most affected region, is common, due to a large number of grouped sebaceous glands, which leads patients to seek care because of health concerns or aesthetic reasons.^5^

Histopathology reveals sebaceous glands as found in the skin.^4^

### Leukoedema

It is a condition of the oral mucosa often found in black-skinned individuals, although it is not exclusive in this population. Leukoedema is an anatomical variation of the oral cavity (Fig. 1B). It mainly affects the buccal region, which acquires a grayish-white, milky and opalescent appearance. More rarely, it affects the sides of the tongue. A maneuver that facilitates the diagnosis is to make it disappear by stretching the buccal mucosa; the lesion is once again seen when the maneuver is discontinued.^4^, ^6^

### Fissured tongue

This is a common anatomical variant, characterized by a varied number of grooves that affect the dorsum of the tongue with variable depth (Fig. 1C). Some individuals have only one longer central fissure. It can be seen in both children and adults, with prevalence increasing with age. It is a classic but less important sign of Melkersson-Rosenthal syndrome.^7^ Moreover, there is a strong relationship between fissured tongue and geographic tongue, with several individuals displaying both conditions.^4^ The patient should be advised to brush the tongue during oral hygiene.^4^, ^7^

### Geographic tongue (erythema migrans, geographic mucositis)

Geographic tongue is characterized by areas without papillae with a migratory pattern, modifying its design daily (Fig. 1D). Sometimes, these depapillated areas have more keratinized edges than the rest of the tongue, with histopathology similar to that of psoriasis. This histopathological finding of the edges of some lesions makes some authors attribute a psoriatic etiology (a disease characterized by fixed and thickened/hyperkeratotic plaques) to a migratory and atrophic disease. As the only symptom, sensitivity to citrus or spicy foods may occur in the depapillated areas in some individuals. A condition of unknown etiology, it affects between 1% and 3% of individuals. It may affect other areas of the oral mucosa, such as the buccal mucosa, the palate, the labial mucosa, and the ventral tongue.^4^

### Coated tongue/black hairy tongue

**Figure 2:**
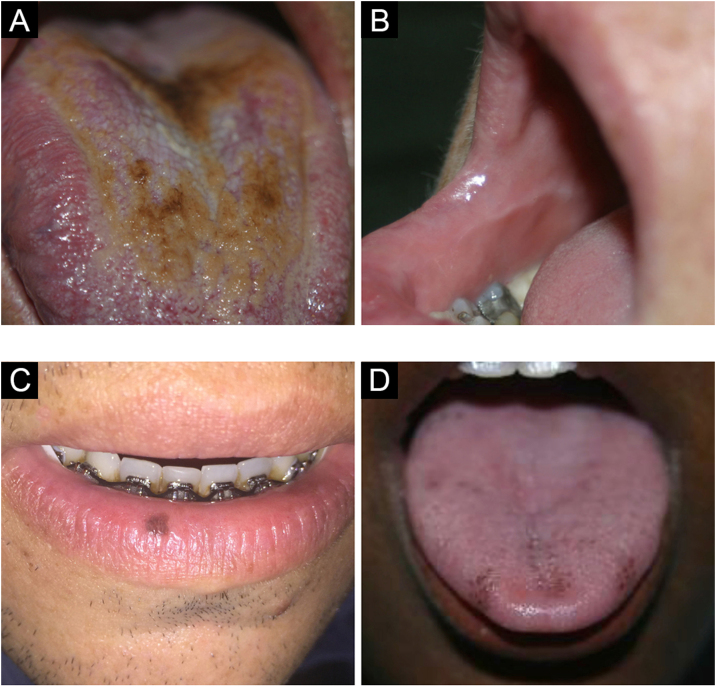
(A) Hairy black tongue. (B) Linea alba. (C) Oral melanotic macule. (D) Physiological hyperpigmentation

It occurs due to the accumulation of keratin over the filiform papillae on the dorsum of the tongue, which become more elongated, giving it a hairy appearance (Fig. 2A). It is more frequent in smokers, people with poor oral hygiene, debilitated patients and individuals with a history of head and neck radiotherapy. It represents an increase in the production of keratin or a decrease in its normal desquamation. When there are no hairy projections, it is called coated tongue. It may have a yellowish, brown, or black color due to the presence of pigment-producing bacteria.^8^, ^9^

### Linea alba/occlusal line

The linea alba is an alteration in the buccal mucosa, associated with pressure or suction trauma between the vestibular surfaces and the teeth, in the occlusal region (Fig. 2B). Clinically, a raised, white line, usually bilateral, varying in prominence, is observed.^4^ Histopathology of this type of lesion shows hyperorthokeratosis covering normal mucosa. When the same raised line occurs with normal color, not white, it is called an ‘occlusal line’.^4^, ^7^

### Oral melanotic macule

The oral melanotic macule is usually single, well delimited, brownish or black in color (Fig. 2C). It occurs mainly in the lower lip semi-mucosa when it can also be called lip melanotic macule; sun exposure is questioned as a causal factor by some authors. It can also affect the buccal mucosa, gingiva and palate. It is more frequent in women, with no predilection for any age group. On histopathology, it is characterized by increased melanin production with morphologically normal basal layer melanocytes.^10^, ^11^

### Physiological pigmentation

Physiological pigmentation is characterized by circumscribed areas, either single or multiple, of hyperpigmentation on the oral mucosa, usually in people with high phototypes, affecting mainly the gingiva, buccal mucosa, and palate (Fig. 2D). It must be differentiated primarily from drug-induced pigmentation, such as due to minocycline, which causes blue-gray discoloration by drug metabolites deposition.^4^

### Palatine and mandibular torus

**Figure 3:**
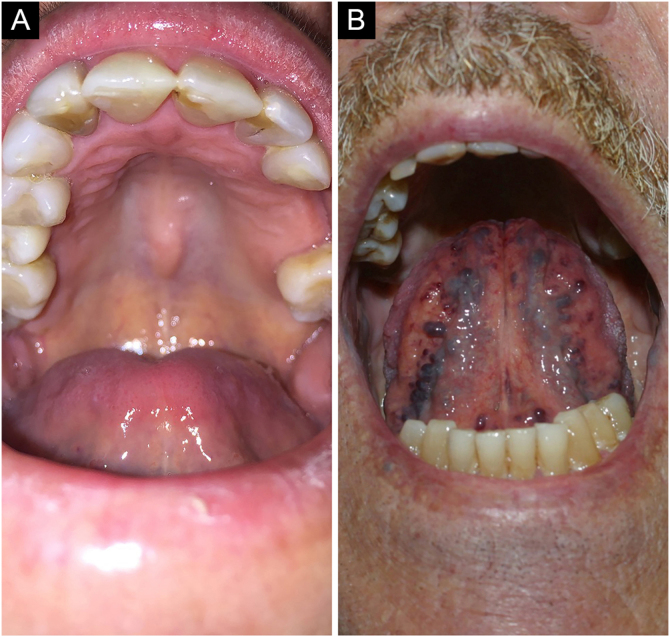
(A) Palatine torus. (B) Lingual varicose veins

Palatine torus (plural form: tori) is a common exostosis that occurs on the roof of the oral cavity. It manifests as an elevated bone mass covered by normal mucosa arranged along the suture in the midline of the hard palate, and may have a flat, nodular, or lobular appearance (Fig. 3A). They are usually small and asymptomatic masses, smaller than 2 cm. Inspection and palpation of the lesions are sufficient to characterize them.^4^

Torus mandibularis or mandibular torus is a common exostosis that appears as a bony protuberance along the mandible, above the mylohyoid line, in the premolar region. Bilateral involvement occurs in 90% of cases and usually consists of single nodules, which may also be multiple. Its prevalence is lower than that of a palatine torus.^4^ Most cases are diagnosed clinically.^4^

### Lingual varicose veins

Varicose veins are abnormally dilated and tortuous veins (Fig. 3B). They are very common in the elderly and rare in children, suggesting that this condition is an age-related degeneration due to the loss of the connective tissue that supports the vessels. It is estimated that they occur in 2/3 of the population over 60 years of age. It is not associated with systemic diseases. Classically, they present as multiple bluish or purplish elevations, most commonly on the ventral part of the tongue.^4^

## Traumatic lesions

### Epulis fissuratum/fissured epulis

**Figure 4:**
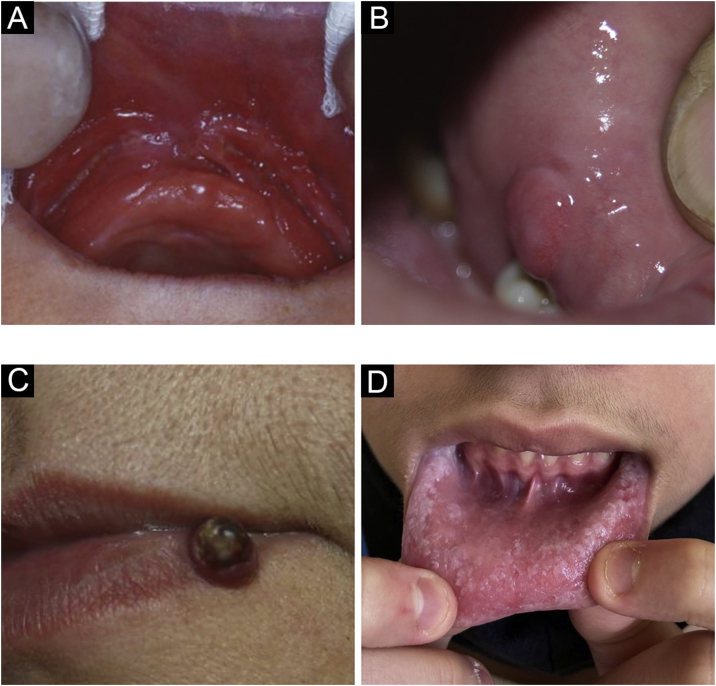
(A) Fissured epulis. (B) Traumatic fibroma. (C) Pyogenic granuloma. (D) *Morsicatio buccarum*

It is a hyperplastic lesion juxtaposed and parallel to the prosthesis attachment area, on one side or both sides, with a central fissure corresponding to the prosthesis attachment area (Fig. 4A). The redundant tissue is usually firm and may be fibrous; some lesions may resemble pyogenic granuloma. It may occur in the maxilla or the mandible. On histopathology, there is hyperplasia of the fibrous connective tissue.^4^

### Irritation or traumatic fibroma

Gingival fibroma is a reactive hyperplasia of fibrous connective tissue in response to trauma or irritation. It is a common disease, most often producing sessile and occasionally pedunculated lesions. The most frequent location is on the biting line, but it can occur in any area of the oral mucosa. The lesions have a smooth surface similar in color to the surrounding mucosa (Fig. 4B). Some may become hypochromic from keratinization due to repeated trauma. The lesions are asymptomatic and usually less than 1.5 cm. On histopathology, they are a mass of fibrous connective tissue covered by stratified squamous epithelium.^4^

### Pyogenic granuloma

Pyogenic granuloma is a proliferative lesion that occurs after minimal trauma. It consists of a tumor growth of non-neoplastic nature, caused by an exaggerated reaction of granulation and vascular tissue, with a tendency to bleeding (Fig. 4C). It is the most frequent oral tumor in children and young people. In addition, pregnant women classically develop these diseases. The gingival region is the most frequently affected area, but other areas can be affected, such as the lips or tongue. It can sometimes be confused with gingival hyperplasia.^7^

### Irritant/frictional leukokeratosis

A most frequent cause of oral white (or leukoplakia-like) lesions. It represents the thickening and consequent keratinization of the mucosa by repeated trauma, the equivalent of a callus formation. Clinically, bilateral lesions are seen on the buccal mucosa, but also on the lateral border of the tongue and even on the lips. Thick white areas are identified, sometimes interspersed with erythema, erosions and purpura. Eventually, the patients describe that they can manipulate the lesion. One must be attentive to teeth or dental arch irregularities, and orthoses or prostheses as possible causative agents, both chronic and acute.^4^

### Morsicatio buccarum

This term is used to designate repetitive biting trauma, causing irregular keratinization of the buccal mucosa that becomes white, with a shredded appearance (Fig. 4D). It is a specific type of frictional leukokeratosis. It can be unilateral or, more often, bilateral. A similar picture may occur on the sides of the tongue or lip mucosa and it is associated with anxiety or stress. On histopathology, it presents with irregular keratinization, reproducing the clinical appearance.^4^

### Mucocele and ranula

**Figure 5:**
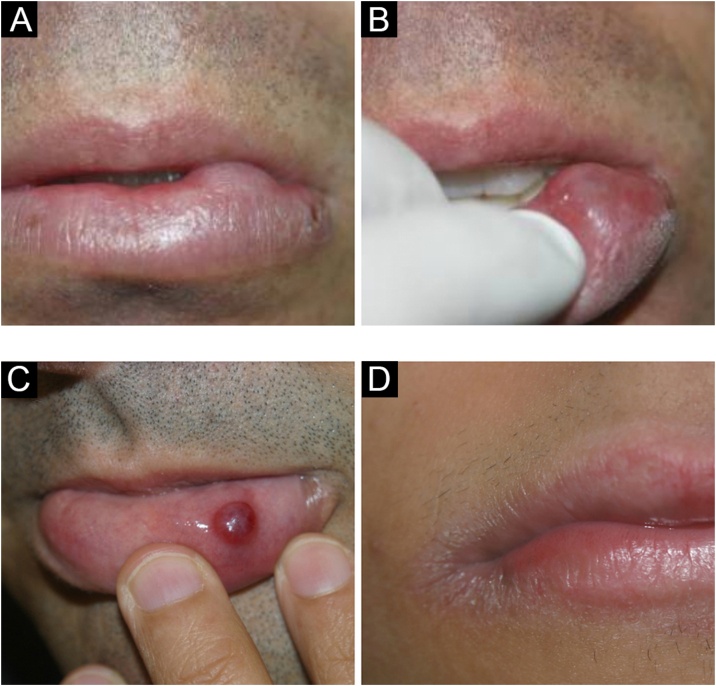
(A and B) Mucocele. (C) Hemorrhagic mucocele. (D) Exfoliative cheilitis

These are common lesions of the oral mucosa, resulting from the rupture of the minor salivary gland duct and consequent spillage of mucus into adjacent tissues.^4^ They most frequently occur on the lower lip mucosa by biting (Figs. 5 A–C). Unlike salivary gland cysts, mucocele does not have an epithelial lining and is therefore not a true cyst.^12^, ^13^, ^14^

The mucin extravasated beneath the mucosal surface sometimes imparts a translucent blue hue. It is not uncommon for it to have hemorrhagic content. On the other hand, very superficial lesions have a vesicular aspect. Pathologists should be aware of this lesion and not confuse it on histopathology with vesiculobullous disorders, especially mucous membrane pemphigoid.^12^, ^13^

Ranulas are mucoceles that occur on the floor of the mouth, involving the major salivary glands (sublingual and rarely submandibular). Clinically, a translucent mass, which may also be bluish, is observed on the floor of the mouth, resembling a “toad belly”, hence the name ranula.^15^

### Exfoliative cheilitis

Persistent desquamation of the labial semimucosa and/or the skin of the lips, caused by the habit of licking the lips. It is also called lip licking (Fig. 5D). There is a predominance in young people, less than 30 years old. The lesions begin with dryness of the skin and progress to erythema, desquamation and fissuring and may become covered by a yellowish or hemorrhagic hyperkeratotic crust, which may lead to hyperpigmentation of the cutaneous side of the lips.^4^

## Infectious lesions

### Candidiasis

It is the most common fungal infection of the oral cavity, and the main etiological agent is *Candida albicans*. It is worth remembering that this organism can be a component of the normal oral microflora, present in up to 50% of people in the absence of disease. It mainly affects debilitated, immunocompromised individuals. The use of systemic or inhaled corticosteroids is a common cause.^4^

**Figure 6:**
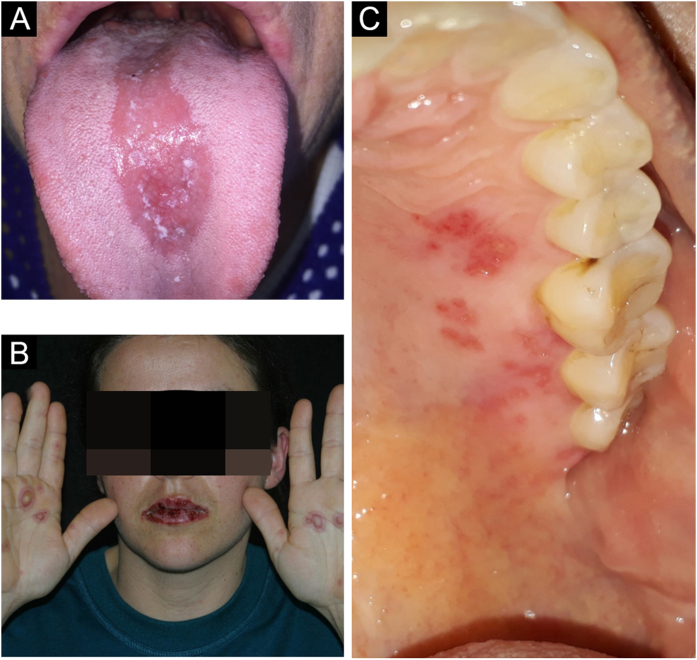
(A) Candidiasis. (B) Labial herpes simplex associated with target lesions on hands. (C) Herpes zoster with unilateral erosions (kindly provided by Prof. Hiram Larangeira de Almeida Jr.)

It presents in different forms: pseudomembranous (the pseudomembranes can usually be removed with gauze, leaving an erythematous, eroded or ulcerated surface); erythematous, multifocal chronic form (atrophy of the papillary center of the tongue and involvement of other areas), chronic atrophic or denture stomatitis (in the support areas of a removable dental prosthesis), angular cheilitis (accumulation of saliva favoring infection) and mucocutaneous (rare, associated with a group of immunological disorders; Fig. 6A). Median rhomboid glossitis or central papillary atrophy (erythematous, well-defined area in the posterior midline of the tongue) is a controversial condition that has already been considered a developmental defect and is probably caused by *Candida*, with improvement not always complete when treated as candidiasis. The presence of dysphagia should lead to the suspicion of esophageal candidiasis.^4^

### Leprosy

Oral lesions are uncommon in tuberculoid and borderline forms, occurring more frequently in the lepromatous form. Sites cooled by the passage of air are the most often affected, with a preference for the palate. It initially presents as firm, sessile, reddish-yellow papules that develop into ulceration and necrosis; with complete loss of the uvula and bone destruction may occur due to local infiltration. Lip involvement can lead to macrocheilia, and maxillary involvement in children can affect dental development.^4^, ^16^

### Herpes simplex

The symptomatic form of herpetic primary infection manifests as gingivostomatitis and usually affects children. Systemic symptoms such as fever, nausea, and irritability are present. It is characterized by vesicles, which rapidly coalesce to form multiple small erythematous lesions that progress to fibrin-covered central ulceration (Fig. 6B).^4^

Recurrence occurs due to viral reactivation and is usually associated with factors such as physical or emotional stress, ultraviolet radiation, local trauma, pregnancy, and events that reduce immunity. Lesions occur at the sites of primary inoculation or adjacent areas; they are more frequent on the lip vermilion. In immunosuppressed patients, recurrences are often more extensive and persistent; there are large areas of erosion or ulceration, sometimes covered by necrotic crust.^4^

In immunosuppressed patients with periorificial ulcerated or necrotic lesions, whether oral, nasal, genital, or anal, it is always suggested to consider the hypothesis of herpes simplex due to its high prevalence in these populations, making the diagnosis very likely in these individuals.^4^

### Herpes zoster

A painful prodromal phase occurs in practically 90% of the cases, with a burning and/or paresthetic feeling; eventually the prodrome manifests as dental pain. Herpes zoster (HZ) oral lesions occur when the trigeminal nerve is involved, extending without crossing the midline, often alongside ipsilateral skin involvement (Fig. 6C). Vesicles progress to ulcerated/aphthous lesions and may coalesce.^17^

A possible complication of HZ infection of the trigeminal or maxillary facial nerve is the development of cranial and peripheral paralysis, such as Ramsay-Hunt syndrome, in which the patient develops Bell's palsy, vesicles in the external auditory canal, and loss of sensation in both anterior thirds of the tongue.^18^

### Focal epithelial hyperplasia

Focal epithelial hyperplasia, also called Heck's disease, has been described in Native American and Inuit populations. The disease is also seen in indigenous groups in South and Central America.^19^ The disease is caused by HPV 13 and HPV 32, associated with a genetic predisposition. No association with malignant lesions has been observed.^20^

It presents as papular lesions that coalesce, acquiring the aspect of “pavement stones”, generally asymptomatic, with a smooth surface. The diagnosis involves clinical identification of the lesions, associated with histopathological analysis. Molecular biology techniques can be employed to ascertain the presence of the HPV virus.^20^

### Histoplasmosis

Most oral lesions occur in the disseminated form of the disease and can affect any area of the oral cavity.^21^ They usually occur as multiple painful verrucous ulcerations, deep ulcers surrounded by infiltrative borders with erythematous or white areas and irregular surfaces, as well as hardened and irregular nodular lesions accompanied by local lymphadenopathy, mimicking other infectious diseases or malignant tumors. The most commonly involved sites in the oral cavity are the tongue, palate, oral mucosa, gingiva, and pharynx.

The differential diagnosis should include squamous cell carcinoma, hematological malignancies, tuberculosis, other deep fungal infections, oral lesions seen in Crohn's disease, necrotizing sialometaplasia of the palate, and chronic traumatic ulcers.^21^, ^22^

### Mucocutaneous leishmaniasis

Mucosal involvement is relatively rare and results from the hematogenous or lymphatic spread of amastigotes from the skin to the nasal, oropharyngeal, laryngeal, or tracheal mucosa.^23^

When it affects the oral mucosa, the disease becomes destructive or ulcerovegetative and granulomatous, accompanied by coarse granules and deep grooves normally associated with painful symptoms, deglutition difficulties, sialorrhea, fetid odor, and bleeding. In the oral cavity, the sites most often affected by these lesions are the lips, hard palate, soft palate, and uvula, whereas lesions of the alveolar, tongue, tonsils, and retromolar regions are rare and are mainly associated with immunosuppression.^24^

### Paracoccidioidomycosis

**Figure 7:**
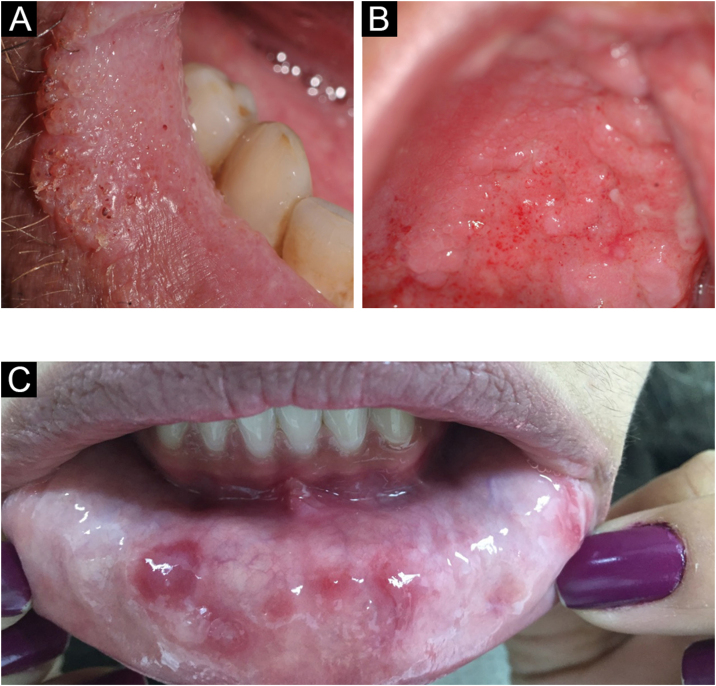
(A) Labial moriform lesion (kindly provided by Prof. Sílvio Alencar Marques). (B) Moriform stomatitis (kindly provided by Prof. Sílvio Alencar Marques). (C) Syphilis

The oral manifestation of paracoccidioidomycosis is extremely important for the diagnosis of the disease; it is the main anatomical area for confirmatory biopsy.^25^ Spread to oral and nasal mucosa usually occurs after initial lung involvement.^26^ The oral, pharyngeal, and laryngeal mucosa are involved in up to 70% of adult patients.^27^ In general, the lesions present as granulomatous and erythematous hyperplasia, interspersed with hemorrhagic spots, called moriform stomatitis (Figs. 7 A and B), followed by ulceration. The gingiva and the palate are the most affected sites.^28^

### Syphilis

Likewise in the skin, syphilis in the oral mucosa also shows a huge variety of presentations at different stages of the disease, making it a diagnostic challenge in clinical practice.^7^

Primary syphilis manifests its chancre as a single, deep ulcer with an erythematous, purplish or brownish base and irregular, raised borders, usually accompanying cervical lymphadenopathy (Fig. 7C). In most cases, the lesion appears on the lips – in men, mainly on the upper lip, and in women, on the lower lip – and more rarely on the tongue. Important differential diagnoses at this stage include traumatic ulcers and squamous cell carcinoma.^29^

In secondary syphilis, macular syphilides stand out, which manifest as small reddish plaques on the hard palate, which are superficial ulcers of the mucosa, rich in treponema, covered by whitish exudate, and flat condyloma, similar to the ones that occur in the skin.^29^

In tertiary syphilis, the most common lesion is the gumma, in the oral mucosa as well as in the rest of the body, with a hardened, nodular appearance that later ulcerates, with great tissue destruction.^29^

### Viral/HPV wart

Warts viruses belong to a large group (>100) of DNA viruses, the papillomaviruses or HPVs. Some subtypes are often found in the oral or genital cavity such as HPV-6, -11, -16, -18. Clinically, they present as raised white or pinkish papules, eventually filiform, on the palate, gingiva, tongue, and labial mucosa. Progression to verrucous carcinoma can occur, also known as oral florid papillomatosis. The diagnosis of HPV can be confirmed on histopathology and is characterized by papillomatosis, parakeratosis, hyperkeratosis, and koilocytosis.^4^

## Inflammatory/Miscellaneous

### Recurrent oral aphthous ulcers

Recurrent aphthous stomatitis is the most common affection of the oral mucosa, characterized by the appearance of ulcerative lesions in any region of the buccal mucosa, which may vary in size, number, and distribution. The etiology is unknown; the lesions may also be triggered by a bite and carriers report their emergence or aggravation related to their emotional state. The disease is divided into three types: minor recurrent aphthous stomatitis, major recurrent aphthous stomatitis, and herpetiform aphthous lesions.^30^, ^31^

The minor form is the common aphthous lesion. They are circular or shallow oval lesions and usually measure up to 5 mm in diameter. They have a grayish-white pseudomembrane, surrounded by an erythematous halo. They occur in the labial, buccal mucosa and floor of the mouth. They disappear without leaving a scar, usually within 7 to 10 days. In a Brazilian population study, in which one of the authors participated, the prevalence of recurrent aphthous lesions in 18-year-old males in the city of Pelotas, state of Rio Grande do Sul, showed a prevalence greater than 20%.^32^

The major form is more rare, known as “Sutton's ulcer”, and usually appears after puberty. These are larger lesions, larger than 1 cm, and very painful; lasts 20 to 30 days and may leave a scar.^4^

The third variation is the herpetiform aphthous ulcer. It is rare, characterized by multiple smaller lesions, ranging from 1 to 3 mm in diameter. Lesions may converge to form larger plaques. They can affect any region of the oral cavity.^4^

Some diseases have aphthous lesions among their manifestations, such as Behçet's disease, cyclic neutropenia, and PFAPA syndrome (periodic fever, aphthous stomatitis, pharyngitis, and adenitis syndrome).^4^

### Hemorrhagic bullous angina

**Figure 8:**
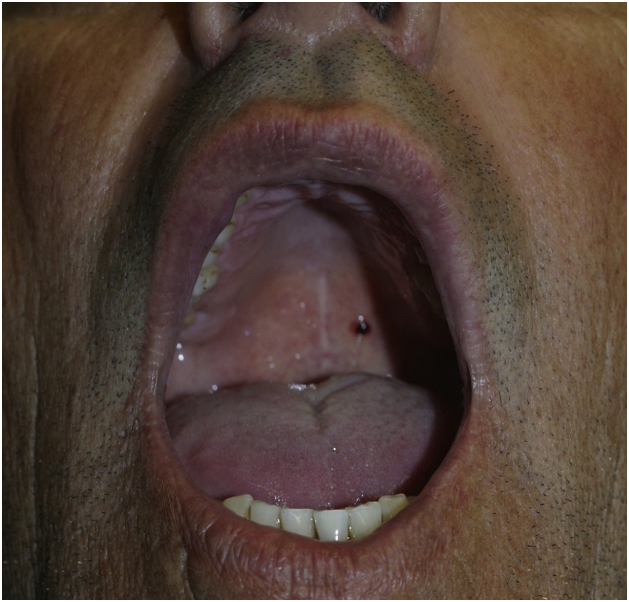
Hemorrhagic bullous angina

Hemorrhagic bullous angina is an uncommon, benign subepithelial disease, which consists in the appearance of an hemorrhagic bulla usually on the palate, measuring 2 cm or larger, which soon ruptures (Fig. 8). Patients may be surprised by an oral hemorrhage while sleeping, due to the ruptured bulla. Some individuals report trauma with food or burning from hot food, but many do not report any trauma. After the rupture, the lesion heals within a few days without leaving a scar.^33^, ^34^ The use of inhaled corticosteroids is an important risk factor for this condition.^35^

### Uremic stomatitis

Uremic stomatitis is a rare disorder related to severe complications of kidney disease. It can manifest itself in four different forms: ulcerative (it is the most common and appears as an ulcerated and erythematous lesion on the mucosa), hemorrhagic (bleeding, mainly in the gingiva), hyperkeratotic (the least common form, occurring in cases of renal failure of long-standing) and erythemato-pultaceous (pseudomembrane formation, usually in patients with controlled renal disease).^36^, ^37^ It may mimic oral hairy leukoplakia. The diagnosis is mainly based on clinical history, oral examination, and laboratory tests. Histopathological findings are non-specific.^36^, ^37^

### Orofacial granulomatosis and Melkersson-Rosenthal syndrome

Orofacial granulomatosis is an uncommon inflammatory disease that affects the soft tissues. The labial region is the most affected. There is infiltrative and persistent edema, and disfiguring fibrosis of the lips and face may occur.^38^, ^39^ Lip involvement alone is called granulomatous (Miescher's) cheilitis. The possibility of associated Crohn's disease or sarcoidosis should be evaluated. Melkersson-Rosenthal syndrome occurs when granulomatous cheilitis is associated with facial palsy and fissured tongue.^40^

On histopathology, there are non-caseous subepithelial granulomas, epithelial hyperplasia, perivascular aggregation of lymphocytes, and an inflammatory infiltrate.

### Lichen planus

It is a chronic inflammatory disease that affects the skin and mucous membranes. Many patients have only oral lichen planus. It is more common in women and its prevalence increases with age. It may manifest in patients with hepatitis C. It has an autoimmune character and, although extremely rare, malignant transformation has been reported.^4^

**Figure 9:**
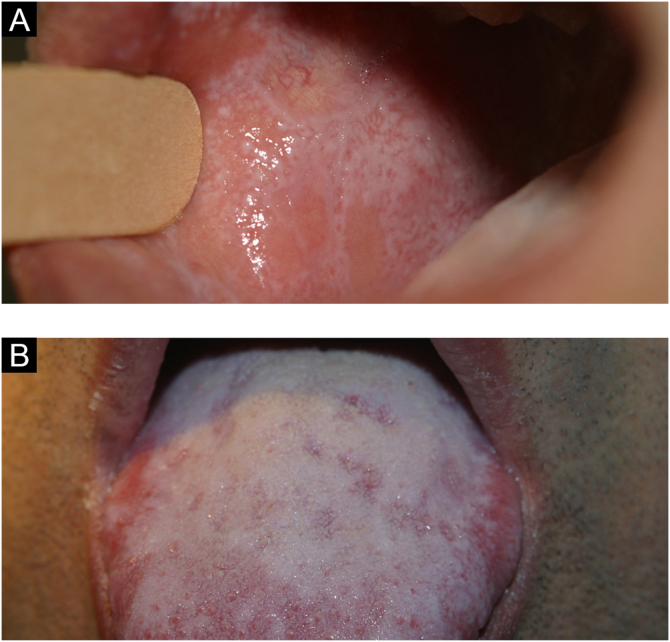
(A) Lichen planus (oral mucosa). (B) Lichen planus (tongue)

The lesions are usually asymptomatic. They appear as reticular areas of fine white striae with a lacy appearance or as white plaques of varying sizes, the dorsum of the tongue being one of the most affected sites (Fig. 9 A and B). It can affect the alveolar ridge, the gingiva, and the palate. Some forms of lichen planus can cause discomfort and pain in patients, such is the case in erosive forms.^4^

An important clinical aspect that also occurs in pemphigus vulgaris and cicatricial pemphigoid is exfoliative gingivitis. The anatomopathological study is useful to exclude other conditions, such as lupus erythematosus, mainly in the cutaneous form, leukoplakia, and bullous diseases.^4^

### Transient lingual papillitis

**Figure 10:**
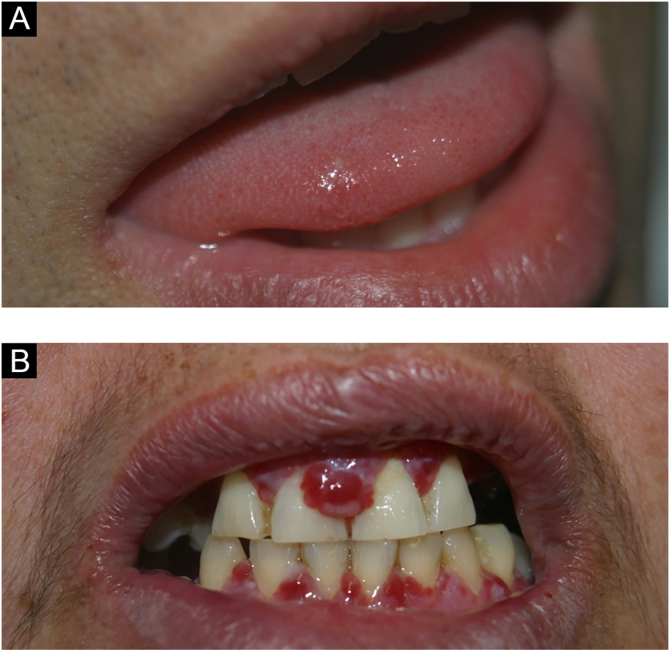
(A) Transient lingual papillitis. (B) Gingival hyperplasia

Transient lingual papillitis or eruptive lingual papillitis are terms used to describe inflammatory hyperplasia of one or several fungiform papillae present on the tongue. The picture is acute and transient. It is a clinical diagnosis and histopathology is not necessary. It manifests as erythematous or whitish papular elevations, painful or not, of about 1 mm on the tongue, which generally disappear within a few hours or days (Fig. 10A). A keratotic more persistent variation may occur. The pathogenesis is unknown, and some patients report its onset with stress.^41^, ^42^

It is an extremely common condition and patients probably confuse it with aphthous lesions, another common condition that usually does not lead to seeking medical attention. Moreover, it has a transient character, similar to aphthous lesions and it is likely to be overlooked by health professionals and, consequently, rarely documented.^43^ In the south of the United States, it is known by the popular name of “lie bumps”.

### Angular cheilitis

Angular cheilitis is an inflammatory reaction, presenting with erythema and maceration of the corners of the mouth. There may be fissures, usually painful ones, as well as crusts, desquamation, and even ulcerations.

Predisposing factors for this condition are advanced age and poor dentition which can lead to the fall of the oral commissures, favoring angular cheilitis. Oral candidiasis and secondary bacterial infections are frequently seen in association with angular cheilitis.^7^, ^44^

An erosive macerated appearance and/or associated with pseudomembranes is suggestive of superimposed candidiasis. Meliceric crusts suggest streptococcal infection.^44^

Secondary syphilis may manifest with an appearance similar to that of angular cheilitis and should be considered in the differential diagnosis.

### Necrotizing sialometaplasia

It is an uncommon inflammatory reaction of unknown cause, locally destructive, usually affecting the minor salivary glands, which can mimic squamous cell or mucoepidermoid carcinoma, both clinically and on histopathology.^45^

Lesions are characterized by non-ulcerated edema accompanied by paresthesia or pain. After two or three weeks, the affected area simply sloughs off, leaving a crateriform ulcer. At this stage, the pain disappears. Patients report that part of the palate simply fell off. It most commonly occurs in the minor salivary glands located on the hard palate. Healing occurs in five to six weeks.^45^

### Burning mouth syndrome (stomatodynia)

Burning mouth syndrome is characterized by a chronic burning sensation in clinically healthy oral mucosa. It most often affects the anterior 1/3 of the tongue (glossodynia or glossopyrosis), but also affects the lips, gingiva, and other parts of the oral cavity.^46^

This disorder is probably a psychiatric one (cancerophobia is reported in 20% of the patients), whether due to obsessive, hallucinatory/psychotic (the most frequent), or paranoid disorders. The manifestations that accompany this disorder are very frequent and curious, such as the sensation of thick saliva, the sensation of gingival or labial swelling, the sensation of foam in the mouth, paresthesia and an endless number of disconnected complaints, or perceptions of alterations that are not seen by the examiner.^47^ Alteration in taste is occasionally reported and, very rarely, loss of taste. The degree of patient suffering is usually important and, in some, even a desperate situation. There are reports of an association with vulvodynia or scrotodynia.^48^

## Drug/allergy reactions

### Cinnamon stomatitis

It means oral contact dermatitis caused by cinnamon products. The clinical presentation of cinnamon stomatitis varies and includes lichenoid erosions, leukoplakia-like patches, gingival erythema, exfoliation, and a leukoedema-like appearance of the mucosa. Patients usually complain of mild pain, pruritus, and a burning sensation.^49^

### Gingival hyperplasia

Drugs are a common cause of abnormal growth of gingival tissues.^50^ Cyclosporine, phenytoin, and nifedipine are strongly associated with this manifestation, reaching an approximately 50% prevalence rate related to phenytoin use.^51^ Tissue enlargement originates in the interdental papillae and spreads across the tooth surface (Fig. 10B). In the absence of inflammation, the gingiva has a normal color and texture. Friable areas resembling pyogenic granuloma may be present. Other causes of gingival hyperplasia are pregnancy and, more rarely, adolescence.^4^

### Bisphosphonate-induced jaw osteonecrosis

Bisphosphonate-induced osteonecrosis is characterized by an area of bone exposure in the maxilla or mandible. In most cases, necrotic bone exposure is observed, ranging from a few millimeters to larger areas, which may be asymptomatic. The reported symptoms are bone pain and changes in tooth mobility. Osteonecrosis is more common in the mandible than in the maxilla, mainly involving areas with less thick mucosa. Radiological alterations can be identified.^52^

## Bullous diseases

### Pemphigoid of the mucous membranes

Desquamative gingivitis is typical, characterized by gingival detachment, erythema, and erosion, but ulcerated and eroded lesions can also be found on the palate. It usually affects women around the sixth decade of life. The bulbar and palpebral conjunctivae are frequently affected, causing morbidity that can lead to blindness.^53^

### Pemphigus vulgaris

Pemphigus is a group of bullous autoimmune diseases, where there is loss of adhesion between cells. Autoantibodies against desmogleins 1 and 3 (anti-Dsg1 and anti-Dsg3) occur.^54^

**Figure 11:**
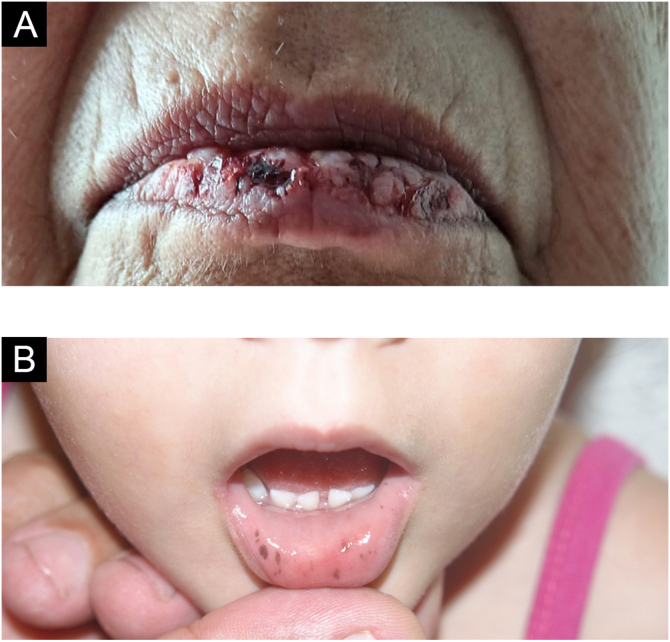
(A) Lip erosions in mucous pemphigus vulgaris (kindly provided by Prof. Hiram Larangeira de Almeida Jr.). (B) Peutz-Jeghers syndrome

Skin involvement can be localized or generalized. Most patients develop flaccid bullae, which rupture at the slightest trauma, leaving eroded areas that bleed easily over normal or erythematous skin. The oral cavity is most frequently affected and most often the initial site of the disease,^54^ with the buccal and palatal mucosa being the most affected sites.^7^ Erosions may be the only oral clinical findings, as the bullae rupture easily (Fig. 11A).^7^

Desquamative gingivitis may occur. Other types of mucosa may be involved, including the conjunctiva, nasal mucosa, pharynx, larynx, esophagus, vagina, penis, and anus.^7^

Intercellular deposits of IgG and C3 are seen on direct immunofluorescence of skin or mucosa. Detection of anti-Dsg1 (mucocutaneous PV) and anti-Dsg3 (mucosal PV) IgG autoantibodies by ELISA occurs in more than 90% of the patients.^7^

### Paraneoplastic pemphigus

It usually presents as a disease that is difficult to control, but improvement occurs with the treatment of the associated neoplasm. Oral involvement is the most common.^55^ The tongue is characteristically involved, but the nasopharyngeal mucosa may also be affected.^56^ Chronic, erosive, progressive, and painful mucositis often occurs and may be the cause of malnutrition due to eating difficulties.^57^

## Genodermatoses

### Cowden's disease or multiple hamartoma syndrome

It has a variable cutaneous clinical picture, from small papules on the face and gingiva to isolated cutaneous tumors. Almost all patients have skin lesions, which usually appear during the second decade of life. It is caused by a mutation in the PTEN phosphatase gene. Multiple facial trichilemmomas, multiple oral papules, and palmoplantar hyperkeratosis form the triad; two of these findings are necessary for the diagnosis. It is associated with benign and malignant neoplasms of the breasts, ovaries, and thyroid.^4^, ^7^

### Spongy white nevus

Rare autosomal dominant condition, characterized by the presence of white, rough and diffuse plaques on the oral mucosa, with a predilection for the buccal mucosa, followed by the ventral surface of the tongue. It more often affects females and has a very varied size and distribution. Extraoral locations such as the vagina, rectum, esophagus and nasal mucous membrane can also be sites of this manifestation. Histopathological findings are characteristic. A family history leads to a definitive diagnosis.^58^

### Fabry disease

Fabry disease, Anderson-Fabry disease, or diffuse corporal angiokeratoma, is an X-linked recessive disease of sphingolipidosis caused by a deficiency of lysosomal hydrolase, or alpha-galactosidase A. It leads to the accumulation of glycolipids in lysosomes. Angiokeratomas are the most common cutaneous signs of this disease, although they are nonspecific.^59^ Telangiectasias are reported to be the second most common cutaneous symptom and are found on the face, lips, oral mucosa, and photoexposed areas.^60^ With manifestations since childhood, it is characterized by hypohidrosis, paresthesia, acral neuropathic pain, with renal, ocular, gastrointestinal, and cardiac alterations, and a predisposition to stroke.

### Pachyonychia congenita

This is an autosomal dominant genodermatosis, characterized by exuberant palmoplantar callosities, mainly plantar, and deforming ungual dystrophies from birth or the neonatal period. Mutations in the genes that encode keratin are responsible for this disease.

Plantar lesions are characteristically painful on walking, probably due to the formation of bullae under the hyperkeratotic areas. Thick white plaques are mostly seen on the sides of the tongue and occur in patients who carry the Keratin 6a (KTR6A) mutation. Mucosal involvement may occur in the larynx, leading some patients to experience hoarseness and dyspnea.^4^

### Peutz-Jeghers syndrome

Peutz-Jeghers syndrome is a rare, autosomal dominant inherited disorder caused by mutations in the STK11 tumor suppressor gene (also known as LKB1). It is characterized by perioral and mucocutaneous pigmentations, gastrointestinal polyposis, and an increased risk of cancer in adulthood.^61^

Pigmentations are seen in approximately 95% of the patients and constitute an early clinical sign, before any gastrointestinal symptoms. The lesions are flat, grayish-blue and vary in size between 1 and 5 mm; they are mainly seen in the perioral region, labial semimucosa and intraorally (Fig. 11B). Pigmented palmoplantar, perianal, and perigenital lesions may also occur. They are usually darker and more clustered than ephelides. Palmoplantar lesions are present in 50% of patients.^1^, ^4^

Peutz-Jeghers syndrome differs from Laugier-Hunziker syndrome in that the latter does not show intestinal polyposis or associated neoplasms.^62^, ^63^

### Hereditary Hemorrhagic Telangiectasia

It is a rare systemic fibrovascular dysplasia, also known as Rendu-Osler-Weber syndrome, which is characterized by a defect in the formation of blood vessel walls, making them more susceptible to trauma or spontaneous ruptures. The most common symptom is frequent epistaxis, seen in about 80% of the patients. Macular telangiectasias can be seen on the mucocutaneous surface, and may occur on the face, lips, ears, nose, tongue, hands, trunk and feet. Other systemic, pulmonary, brain, and gastrointestinal symptoms may be present.^64^

The syndrome is diagnosed by finding at least three of these criteria: telangiectasias of the face, hands, and oral cavity, frequent epistaxis, arteriovenous malformations with visceral involvement, and family history.^65^

## Neoplastic diseases

### Squamous cell carcinoma

Squamous cell carcinoma represents more than 90% of all intraoral malignancies, with an increased risk in older age, especially in men. The cause of squamous cell carcinoma is multifactorial, having both intrinsic and extrinsic components. The etiopathogenesis of intraoral squamous cell carcinoma is a little different from neoplasm occurring in the lip vermilion; intraoral neoplasia is strongly influenced by smoking, alcohol consumption and syphilis, while in the case of lip vermilion carcinoma, sun exposure plays an important role, just like in the skin. Several intraoral squamous cell carcinomas are documented in association with or preceded by a potentially malignant lesion, mainly leukoplakia. Furthermore, it is known that the proportion of smokers with intraoral carcinomas is two to three-fold higher than in the general population. When combined with alcohol, the use of both substances carries a relative risk of 15% or more for chronic users.^4^, ^7^

At first, pain sensitivity is minimal, which often causes patients to delay seeking care. Oral squamous cell carcinoma has a varied clinical presentation, including the following: exophytic (enlarged, vegetating, papillary, verrucous), endophytic (erosive, ulcerated), leukoplastic, erythroplastic, or leukoerythroplastic.^4^

Leukoerythroplastic and erythroplastic examples represent the early stages, in which there is no ulceration or edema as yet. An exophytic lesion is irregular, usually hardened, ranging from a normal color to red or white, depending on the vascularization and amount of keratin in the tumor. The endophytic pattern, in turn, has a depressed, irregular, ulcerated central area with a “rolling” border of normal, red or white mucosa. Of all intraoral carcinomas, oral floor lesions are the most likely to arise from pre-existing leukoplakia or erythroplakia. In patients with intraoral carcinoma, cervical lymph node involvement is evident at diagnosis in 30% of cases and occult in 10% to 40% of cases.^4^, ^7^

### Verrucous carcinoma (oral florid papillomatosis)

Verrucous carcinoma is a low-grade variation of oral squamous cell carcinoma that typically affects individuals over the age of 55 with a habit of chewing snuff and tobacco.^4^

**Figure 12:**
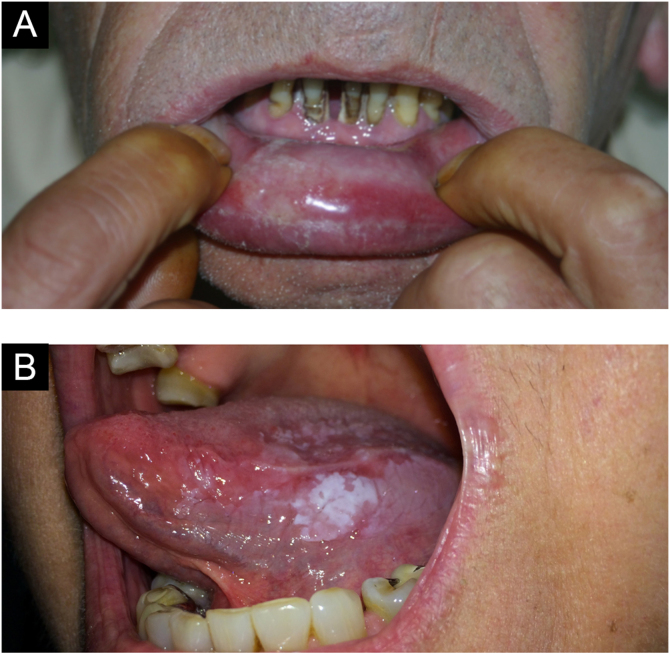
(A) Verrucous carcinoma. (B) Leukoplakia

The lesion appears as a thick, diffuse, well-defined, painless plaque with papillary or verrucous projections on the surface (Fig. 12A). Lesions are white but may appear erythematous or pinkish, depending on the amount of keratin in the tissue.

Without treatment, the neoplasm ends up destroying bone, cartilage, muscle, salivary glands and adjacent structures. Lymph nodes are usually enlarged by a local inflammatory process rather than by lymph node metastases.^4^

The diagnosis of verrucous carcinoma requires adequate incisional biopsy since there is no important epithelial dysplasia to help histopathological confirmation. Clinical exuberance and benign findings on histopathology are the rule.^4^

### Erythroplakia/Leukoerythroplakia

Oral erythroplakia is an uncommon lesion. It shows significant dysplasia early on, corresponding to an “*in situ*” or sometimes invasive squamous cell carcinoma. It occurs mainly in the elderly, more frequently in the soft palate, the floor of the mouth, and the buccal mucosa. It has a smooth and velvety, well-defined appearance, but several other morphological characteristics can be observed, with irregular, red or granular patterns.

When there is concomitant leukoplakia and erythroplakia, it can be called leukoerythroplakia. One must biopsy the erythematous area, because it has the greatest malignant potential.^4^

### Leucoplakia

A well-defined white lesion with a smooth or velvety surface, which may show a verrucous and infiltrated appearance. It can affect any region of the oral cavity, with the buccal mucosa, the lower lip and the tongue the most affected areas. When it occurs in the semimucosa of the lower lip, it is a differential diagnosis for actinic cheilitis.

Leukoplakia corresponds to a clinical term, without correlation with histopathological alterations, which can range from inflammatory to dysplastic features (Fig. 12B). It is a potentially cancerous lesion, often associated with smoking, affecting more adult males. The differential diagnosis, in addition to actinic cheilitis, includes frictional leukokeratosis, lichen planus and squamous cell carcinoma.

Biopsy for histopathology is recommended in cases where typical frictional leukokeratosis is not evident.^4^

### Melanoma

Melanoma is a malignant neoplasm of melanocytic origin. According to the National Cancer Database Report on Cutaneous and Non-cutaneous Melanoma, 91.2% of melanomas arise in the skin, while mucosal melanoma occurs in approximately 1.3%, representing 0.26% of all intraoral cancers. Moreover, at least one in three patients with oral melanoma has a previous personal history of cutaneous melanoma. This type of melanoma, although rare, is more aggressive than its cutaneous counterpart.^4^, ^7^

Oral lentiginous melanoma is usually nodular at the diagnosis, but the initial lesions can be flat, brown, and black in color, with irregular edges. Later on, the macula extends laterally and an exophytic, lobulated growth develops in the vertical growth phase. Approximately 80% of oral melanomas are found in the hard palate or maxillary alveolus. Additionally, approximately 10% of oral melanomas are amelanotic, which may result in diagnostic difficulties, and immunohistochemistry is indicated.^4^, ^7^

### Actinic cheilitis

Actinic cheilitis is a potentially malignant condition of the lower lip vermilion resulting from chronic exposure to UV radiation. Its etiopathogenesis is similar to that of actinic keratosis of the skin, and it also presents a risk of developing into squamous cell carcinoma, especially in smokers and immunosuppressed individuals. This condition usually occurs in individuals over 45 years of age, with a clear predilection for males (male-to-female ratio of 10:1).^4^

The lesion has a slow evolution, often going unnoticed by the patient. Initial clinical findings usually include atrophy (smooth, mottled, pale areas), dryness, and fissures on the lower lip vermilion. As the condition progresses, rough and scaly areas often appear. These areas may thicken, forming leukoplastic lesions.^4^

Many of these alterations are irreversible, but precautions regarding photoprotection should be encouraged. Areas of leukoplakia, thickening, ulceration, or induration should be biopsied for histopathology to exclude carcinoma.

## Conclusions

Oral complaints are frequently found in clinical practice. Medical doctors must familiarize themselves with the most common oral problems, as well as to be able to recognize the anatomical variations of the oral cavity. Moreover, several systemic diseases can be suspected by their mucosal manifestations. This article shows the scope of what can be found in the oral cavity, an easily accessible area for clinical evaluation or sample collection for histopathology. Mouth examination complements clinical examination, and should be routine.

## Financial support

None declared.

## Authors' contributions

Paulo Ricardo Martins Souza: Approval of the final version of the manuscript; design and planning of the study; drafting and editing of the manuscript; collection, analysis, and interpretation of data; effective participation in research orientation; intellectual participation in the propaedeutic conduct of the studied cases; critical review of the literature; critical review of the manuscript.

Leticia Dupont: Approval of the final version of the manuscript; design and planning of the study; drafting and editing of the manuscript; collection, analysis, and interpretation of data; effective participation in research orientation; intellectual participation in the propaedeutic conduct of the studied cases; critical review of the literature; critical review of the manuscript.

Gabriela Mosena: Approval of the final version of the manuscript; design and planning of the study; drafting and editing of the manuscript; collection, analysis, and interpretation of data; effective participation in research orientation; intellectual participation in the propaedeutic conduct of the studied cases; critical review of the literature; critical review of the manuscript.

Manuela Dantas: Approval of the final version of the manuscript; design and planning of the study; drafting and editing of the manuscript; collection, analysis, and interpretation of data; effective participation in research orientation; intellectual participation in the propaedeutic conduct of the studied cases; critical review of the literature; critical review of the manuscript.

Lucas Bulcão: Approval of the final version of the manuscript; design and planning of the study; drafting and editing of the manuscript; collection, analysis, and interpretation of data; effective participation in research orientation; intellectual participation in the propaedeutic conduct of the studied cases; critical review of the literature; critical review of the manuscript.

## Conflicts of interest

None declared.
